# Mechanistic Insights into a Novel Controllable Phase-Transition Polymer for Enhanced Oil Recovery in Mature Waterflooding Reservoirs

**DOI:** 10.3390/nano13243101

**Published:** 2023-12-08

**Authors:** Yong Yang, Xiaopeng Cao, Yanfeng Ji, Ruqiang Zou

**Affiliations:** 1 Shengli Oilfield, SINOPEC, Dongying 257001, China; yangyong@sinopec.com (Y.Y.); caoxiaopeng.slyt@sinopec.com (X.C.); jiyanfeng_001@163.com (Y.J.); 2 School of Materials Science and Engineering, Peking University, Beijing 100871, China

**Keywords:** chemical flooding, controllable phase-transition polymer, mechanistic insights, enhanced oil recovery, mature waterflooding reservoirs

## Abstract

Expanding swept volume technology via continuous-phase polymer solution and dispersed-phase particle gel is an important technique to increase oil production and control water production in mature waterflooding reservoirs. However, problems such as the low viscosity retention rate, deep migration, and weak mobility control of conventional polymers, and the contradiction between migration distance of particle gel and plugging strength, restrict the long-term effectiveness of oil displacement agents and the in-depth sweep efficiency expanding capability in reservoirs. Combined with the technical advantages of polymer and particle gel, a novel controllable phase-transition polymer was developed and systematically studied to gain mechanistic insights into enhanced oil recovery for mature waterflooding reservoirs. To reveal the phase-transition mechanism, the molecular structure, morphology, and rheological properties of the controllable phase-transition polymer were characterized before and after phase transition. The propagation behavior of the controllable phase-transition polymer in porous media was studied by conducting long core flow experiments. Two-dimensional micro visualization and parallel core flooding experiments were performed to investigate the EOR mechanism from porous media to pore level. Results show that the controllable phase-transition polymer could change phase from dispersed-phase particle gel to continuous-phase solution with the prolongation of ageing time. The controllable phase-transition polymer exhibited phase-transition behavior and good propagation capability in porous media. The results of micro visualization flooding experiments showed that the incremental oil recovery of the controllable phase-transition polymer was highest when a particle gel and polymer solution coexisted, followed by a pure continuous-phase polymer solution and pure dispersed-phase particle gel suspension. The recovery rate of the novel controllable phase-transition polymer was 27.2% after waterflooding, which was 8.9% higher than that of conventional polymer, providing a promising candidate for oilfield application.

## 1. Introduction

As oilfields enter the late development stage of high water cut and high recovery, reservoir heterogeneity becomes more serious; the residual oil is dispersed and the injected water enters into the preferential flow channel, resulting in low sweep efficiency and oil recovery rates in mature waterflooding reservoirs. It is predicted that around 60~75% of residual oil still exists in the un-swept zone after waterflooding [1,2,3]. Therefore, recovering the un-swept residual oil is crucial for further enhancing oil recovery. The conventional waterflooding development method fails to greatly enhance oil recovery. How to efficiently recover the potential residual oil and significantly improve its recovery rate remain major challenges for the sustainable development of mature waterflooding reservoirs. Chemical flooding technology can significantly enhance oil recovery by expanding sweep efficiency and improving oil displacement efficiency. Chemical flooding technology can be categorized as follows: polymer flooding [4,5,6,7], surfactant flooding [8,9,10,11,12,13,14,15], nanoparticle flooding [16,17,18,19,20,21,22], surfactant–polymer combined flooding [23,24,25,26,27,28,29,30,31,32,33,34], and heterogeneous phase combined flooding [35,36,37,38,39,40].

Polymer flooding, as one of the most important methods for enhanced oil recovery (EOR), has been successfully applied in a number of mature waterflooding reservoirs in China, such as the Daqing Oilfield, Shengli Oilfield, etc. Generally, the polymer dissolves in water to form an aqueous solution with a continuous phase, which can increase the viscosity of injected water, reduce the water–oil mobility ratio, and improve sweep efficiency [41]. However, due to worsening reservoir heterogeneity and more dispersed residual oil, unrecovered residual oil still exists after polymer flooding. In addition, the EOR efficiency of subsequent surfactant–polymer (SP) flooding after polymer flooding needs to be improved [42]. Therefore, based on SP flooding technology, in recent years, the concept of a heterogeneous phase combined flooding (HPCF) system composed of polymer, branched-performed particle gel, and surfactant was developed and applied in mature reservoirs after polymer flooding in the Shengli Oilfield. Pilot tests have demonstrated that HPCF technology can achieve higher EOR efficiency via a synergistic effect. Specifically, the branched-performed particle gel (B-PPG) can absorb water, swell, and disperse in formation water to form gel particle dispersion. The dispersed-phase gel particles have the characteristics of elastic deformation, migration, and plugging in porous media, which can increase flow resistance and divert the subsequent fluid into the un-swept area.

The continuous-phase polymer solution and dispersed-phase particle gel can expand the swept volume and enhance oil recovery in mature waterflooding reservoirs with high water cut and recovery. However, continuous-phase polymer solutions with increased viscosity and dispersed-phase particle gels have the following technical bottlenecks in field applications: First, continuous-phase polymer solutions increase their viscosity and in-depth migration in porous media, but due to viscosity loss via chemical degradation and shear mechanical degradation, the viscosity retention rate of polymer solutions in a deep reservoir is low, and mobility control is weak. Second, dispersed-phase particle gels have a strong sweep efficiency expanding capability, but there is a contradiction between propagation distance and plugging strength, which leads to an unsatisfactory effect of injection or deep reservoir plugging effect.

To overcome their respective shortcomings, the innovative technical idea of controllable phase-transition polymer flooding that combines the technical advantages of a continuous-phase polymer solution and a dispersed-phase particle gel was proposed to enhance oil recovery significantly. This novel controllable phase-transition polymer, designed and developed successfully at the Shengli Oilfield, can change phase from solid to liquid. The initial state of the controllable phase-transition polymer is a dispersed-phase particle gel state that can deform and migrate in the reservoir. Then, under reservoir conditions, the particle gel dissolves gradually from a solid to a viscous solution. The final state of the controllable phase-transition polymer is in viscous solution form, which has good viscosity-increasing properties.

Thus, in order to reveal the phase-transition behavior, propagation, and enhanced oil recovery mechanism of the novel controllable phase-transition polymer, a series of experiments were systematically conducted to gain mechanistic insights into the enhanced oil recovery for mature waterflooding reservoirs. The molecular structure of the controllable phase-transition polymer was characterized by using Fourier transform infrared spectroscopy. The morphology and rheological properties of the controllable phase-transition polymer before and after phase transition were investigated to reveal the phase-transition mechanism. The propagation behavior of the controllable phase-transition polymer in porous media was studied by conducting long core flow experiments. Two-dimensional micro visualization and parallel core flooding experiments were performed to investigate the EOR mechanism from porous media to pore level. We hope this study can provide new ideas and methods for chemical-enhanced oil recovery in mature waterflooding reservoirs in China.

## 2. Materials and Methods

### 2.1. Materials

The controllable phase-transition polymer used in this study was independently developed by the Exploration and Development Research Institute of the Shengli Oilfield. The phase-transition polymer was prepared as follows: (1) first, the acrylamide (AM), 2-acrylamido-2-methyl propane sulfonic acid (AMPS) monomer and acrylic crosslinking agent were mixed and dispersed via stirring; (2) then, by adjusting the pH value and introducing nitrogen gas, an oxidation-reduction initiator was added to initiate the reaction to synthesize the phase-transition polymer. The salinity of simulated formation water was 19,334 mg·L^−1^. Sodium chloride and calcium chloride used for simulating water preparation were all analytical grade products purchased from Sinopharm Chemical Reagent Co., Ltd. (Shanghai, China). The crude oil used was a simulated oil from a certain block of Shengli Oilfield, with a crude oil viscosity of 60 mPa·s at 68 °C. The molecular weight and hydrolysis degree of the conventional polymer used in this study was 2500 × 10^4^ and 20%, respectively.

### 2.2. Methods

#### 2.2.1. Rheological Characterization

A phase-transition polymer solution with a mass concentration of 2000 mg·L^−1^ was prepared with the simulated formation water by adding predetermined amounts of dry powder via mechanical stirring for 30 min. Then, the prepared phase-transition polymer was deoxygenated and placed in different ampoule bottles in an oven at a simulated reservoir temperature of 68 °C for ageing treatment. At different ageing times, the samples were taken out and their elastic modulus was tested at 68 °C using an Anton Paar (North Ryde, NSW, Australia) MCR302 plate rheometer. The plate spacing, vibration stress, and vibration frequency were set to 0.2 mm, 0.1 Pa, and 1 Hz, respectively. After the controllable phase-transition polymer solution was filtered, the apparent viscosity of the filtrate was tested using an Anton Paar MCR302 rotary rheometer at a temperature of 68 °C and a shear rate of 7.34 s^−1^. The variation law of the elastic modulus and apparent viscosity of the controllable phase-transition polymer was then analyzed.

#### 2.2.2. Chemical Structure Analysis

Fourier transform infrared (FT-IR) spectroscopy was obtained to analyze the chemical structure of the controllable phase-transition polymer. The samples for the FT-IR test were prepared by grinding and pressing the polymer sample with potassium bromide (KBr) powder. The spectra were recorded in a wavenumber range of 4000–650 cm^−1^ for the samples, and the resolution was 4 cm^−1^.

#### 2.2.3. Morphology Characterization

The morphology of the controllable phase-transition polymer at different phase-transition stages was characterized using a Carl Zeiss scanning electron microscope (SEM). The samples were prepared by ageing the phase-transition polymer at 68 °C for different periods. The procedure of lyophilization and SEM observations of samples at different stages were as follows: (1) The samples were prepared by ageing the phase-transition polymer at 68 °C for different periods; (2) Then, the phase-transition polymer samples were transferred to a fresh mica sheet; (3) After freezing in liquid nitrogen for 1 h, the samples were quickly transferred to a lyophilizer, in which the condenser temperature was −80 °C, for 24 h; (4) After freeze-drying and gold spraying treatment, the morphology of the phase-transition polymer at different phase-transition stages was observed via scanning electron microscopy (SEM).

#### 2.2.4. Propagation Behavior of the Phase-Transition Polymer in Porous Media

The propagation behavior of the phase-transition polymer in porous media was investigated by performing sand-pack flow experiments (Figure S1). In this study, sand-pack (Φ2.5 cm × 30 cm) was used with different pressure measuring points located at the injection end (P_1_), at 10 cm (P_2_), and at 20 cm (P_3_) near the injection end. All the flow experiments were performed at 68 °C. The experimental processes are as follows: (1) Sand-packs with different permeabilities were prepared using the wet-packing method. During the filling process, quartz sand and simulated formation brine were alternately added and compacted. The sand-pack pore volume was the volume of used simulated formation brine. (2) Waterflooding was performed by injecting the formation brine at a certain flow rate until the injection pressure was stable followed by the calculation of permeability according to stable injection pressure ∆*P*_wa_. (3) Phase-transition polymer at different ageing stages was injected, and then subsequent waterflooding was conducted. The injection pressures during polymer injection (∆*P*_polymer_) and subsequent waterflooding process (∆*P*_wb_) were recorded. The same injection rate was applied during the polymer injection and subsequent waterflooding process.

Based on the injection pressures at different stages, the resistance factor *F_r_* and residual resistance factor *F_rr,_* which can reflect its propagation and plugging characteristics in porous media, were calculated according to the following equations:

(1) $$F_{r}=\frac{\Delta P_{\mathrm{polymer}}}{\Delta P_{\mathrm{wa}}}$$

(2) $$F_{rr}=\frac{\Delta P_{\mathrm{wb}}}{\Delta P_{\mathrm{wa}}}$$

where ∆*P*_polymer_, ∆*P*_wa_, and ∆*P*_wb_ present injection pressures during polymer injection, stable injection pressure, and injection pressures during the waterflooding process, respectively.

#### 2.2.5. The 2D Glass-Etched Micromodel Flooding Experiment

To reveal the mechanism behind the enhanced oil recovery of the controllable phase-transition polymer flooding at different stages, 2D glass-etched micromodel flooding experiments were performed. The glass-etched micromodel was composed of an etched plate and cover plate, which were attached together and sealed to form an enclosed pore space. An etched plate with heterogeneous pore size distribution was designed, and the size of the micromodel was 4 mm × 4 mm. The 2D visualization experimental apparatus (Figure S2) was composed of a transparent glass-etched micromodel, high precise syringe pump, LED light source, high-resolution camera, and image acquisition and processing system. The high-resolution camera was used to record the flow behavior of the phase-transition polymer in the glass micromodel at different flooding stages. The image acquisition and processing system was used to measure the change in oil saturation and for calculating the oil recovery at different flooding stages. The specific experimental process is as follows: (1) The model was vacuumed for saturating crude oil. (2) Waterflooding was conducted until no oil was produced at the outlet of the model, at an injection rate of 0.001 mL/min. (3) The phase-transition polymer flooding was conducted at an injection rate of 0.001 mL/min. (4) The distribution of oil and water in the micromodel was observed and recorded throughout the entire flooding process. (5) The EOR efficiency of phase-transition polymer was calculated at different flooding stages.

#### 2.2.6. Evaluation of Enhanced Oil Recovery Capability

**Figure 1:**
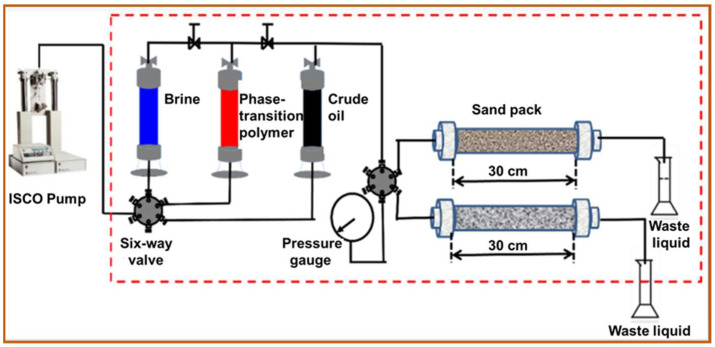
Experimental flow chart of parallel sand-pack flooding.

To simulate the heterogeneous reservoir conditions of the Shengli Oilfield, parallel sand-pack flooding experiments were conducted to investigate the enhanced oil recovery capability of the controlled phase-transition polymer at different stages. The permeabilities of the parallel sand-pack model (φ2.5 cm × 30 cm, Figure 1) were 3000 × 10^−3^ μm^2^ and 1000 × 10^−3^ μm^2^, respectively. The specific experimental procedures are as follows: (1) The sand-pack was filled with different mesh quartz sand using the wet packing method, and liquid permeability was measured at a waterflooding rate of 1.0 mL/min. (2) The wet-packed sand-pack was flooded with crude oil at a rate of 0.1 mL/min until complete oil saturation. Then, the sand-pack was put in the oven at 68 °C for ageing for 48 h after oil saturation. (3) The initial waterflooding was conducted until the water cut reached 95%, at a flooding rate of 0.3 mL/min. Then, a 0.5 PV polymer slug was injected into the sand-packs for chemical flooding. (4) Subsequent waterflooding was conducted until the water cut reached 98%. Then, the flooding experiments were terminated. The injection pressure and volumes of the water and oil produced were recorded at different flooding periods.

## 3. Results and Discussion

### 3.1. Morphology and Chemical Structure of the Phase-Transition Polymer

**Figure 2:**
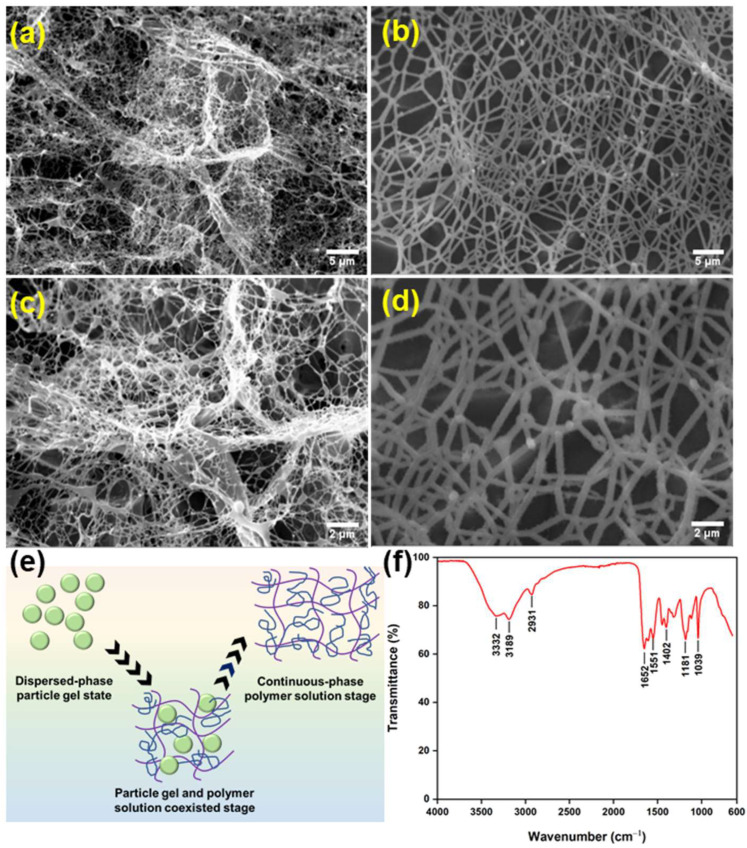
(**a**,**b**) SEM images of the polymer before phase transition; (**c**,**d**) SEM images of the polymer after phase transition; (**e**) scheme of the morphology changes of the phase-transition polymer; and (**f**) FT-IR spectra of this polymer. (**a**,**b**): magnification of 2 k×; (**c**,**d**): magnification of 5 k×.

To reveal the morphology changes of the controllable phase-transition polymer during its phase-transition process, SEM images of the polymer at different phase-transition stages were acquired. As noted in Figure 2a,c, the controllable phase-transition polymer was in a state of dispersed-phase particle gel at the initial stage before phase transition, and a small amount of dense network structure can be observed. With the prolongation of ageing time, the unique weak cross-linking structure of the controllable phase-transition polymer was hydrolyzed and transformed into a continuous-phase state of high viscosity polymer solution. At this stage, the morphology of the controllable phase-transition polymer presented a large number of network structures (Figure 2b,d), which endow excellent viscosity-increasing properties, as supported by the rheological results. Digital images of the controllable phase-transition polymer at different stages provide similar findings (Figure S3). The scheme of the morphology changes of the phase-transition polymer is summarized in Figure 2e.

The FT-IR can characterize the molecular structure of the phase-transition polymer, and the obtained spectrum is presented in Figure 2f. It can be seen that the stretching vibration peaks of the NH_2_ group of the primary amide are at 3332 cm^−1^ and 3189 cm^−1^, respectively. A characteristic absorption peak of -CH_3_ was found at 2931 cm^−1^. The strong and sharp absorption peak at 1652 cm^−1^ is a characteristic absorption peak of carbonyl groups with a lower wavenumber, usually the amide carbonyl absorption peak. Combined with the absorption peaks at 3332 cm^−1^ and 3189 cm^−1^, it can be inferred that the -CONH_2_ group is present in the polymer [43]. Based on the above analysis, the controllable-phase polymer proves to be a class of acrylamide polymer. Peaks at 1181 cm^−1^ and 1039 cm^−1^ are attributed to the symmetric bending and asymmetric stretching vibrations of sulfonic-acid-based -SO_3_^2−^ [44]. Moreover, it is clear that the phase-transition polymer contains a unique functional -OH group and a large amount of -SO_3_^2−^; the monomers acrylamide and 2-acrylamido-2-methyl propane sulfonic acid used to synthesize the polymer contribute to the chemical characteristics.

### 3.2. Rheological Properties of the Phase-Transition Polymer

**Figure 3:**
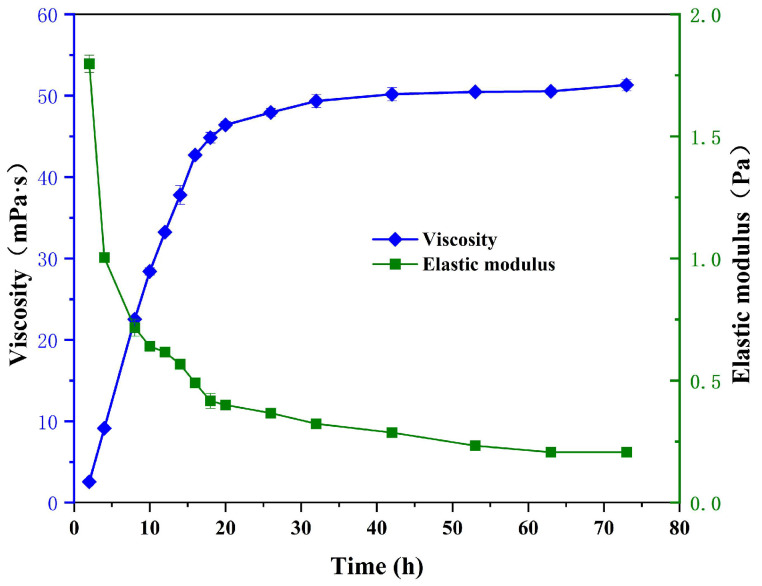
Changes in viscosity and elastic modulus of phase-transition polymer versus time.

According to the rheological property characterization method of the phase-transition polymer during the phase-transition process, the viscosity and elastic modulus of phase-transition polymer versus time are depicted in Figure 3. It can be seen that the controllable phase-transition polymer has the characteristics of high viscoelasticity and a deformable dispersed-phase particle gel at the initial stage; the viscosity of the particle gel suspension is about 1.0 mPa∙s, which indicates that the polymer has not yet been dissolved, and its viscosity is low. This is because the cross-linking group of controllable weak cross-linking agent combines with the carboxyl group in the polymer molecular chain, which greatly reduces the extension of the polymer molecular chain and results in a bound solid dispersed-phase particle gel. The curled molecular structure inhibits the hydration of amide and carboxyl groups, making it difficult to increase viscosity. Meanwhile, the elastic modulus of the solution is about 5 Pa when the phase-transition polymer presents as a dispersed-phase particle gel.

As the ageing time prolongs, viscosity gradually increases. This is because the weak cross-linking structure between polymer molecular chains gradually undergoes hydrolysis, allowing the polymer molecular chains to extend and release amide and carboxyl hydrophilic groups. The hydration effect of the molecular chains gradually increases, and they intertwine to form a network structure, resulting in an increase in viscosity, as reported elsewhere [45]. Additionally, the elastic modulus decreases significantly, which indicates that the sample’s solid-like behavior deteriorates. During the polymer ageing process, the particle gel gradually dissolves in water, forming the intermediate phase when particle gel and polymer solution coexist, leading to a gradually decreased elastic modulus and increased viscosity. When it comes to the viscosity stabilization stage, the viscosity and elastic modulus of phase-transition polymer tends to level off. This can be attributed to the fact that the weak cross-linked structure is completely broken under the ageing effect, and the solid gel particles are almost all dissolved in water and transformed into a continuous-phase aqueous solution.

**Figure 4:**
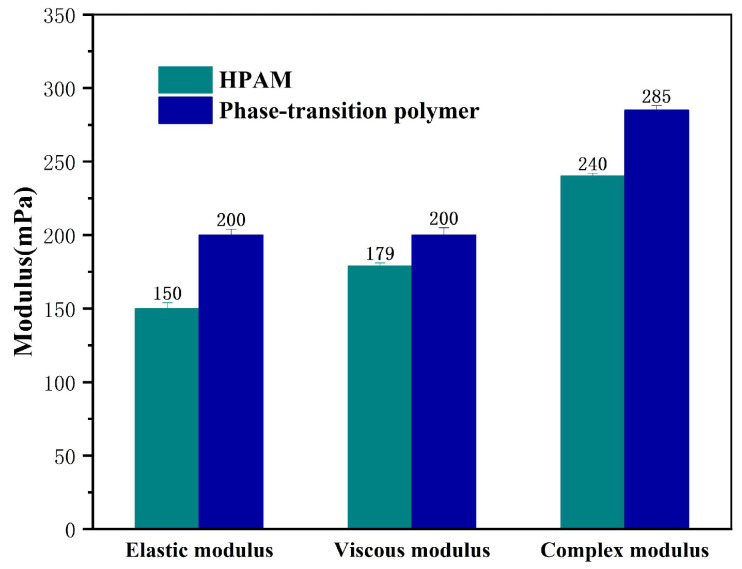
Comparison of viscoelastic modulus of conventional HPAM and phase-transition polymer after phase transition.

The viscoelastic properties of controllable phase-transition polymer in an aqueous solution phase and conventional hydrolyzed polyacrylamide (HPAM) were further investigated and compared (Figure 4). As noticed, the elastic modulus, viscous modulus, and complex modulus of the controllable phase-transition polymer in an aqueous solution phase are slightly higher than those of conventional HPAM. This indicates that under the branching effect of a weak cross-linking structure, the molecular chains of a controllable phase-transition polymer are more stretched, and the intermolecular entanglement is more obvious compared to HPAM. Consequently, the structural and rheological properties of a phase-transition polymer may benefit their functioning in a targeted application, i.e., enhanced oil recovery in mature waterflooding reservoirs.

### 3.3. Propagation Behavior of the Phase-Transition Polymer in Porous Media

**Figure 5:**
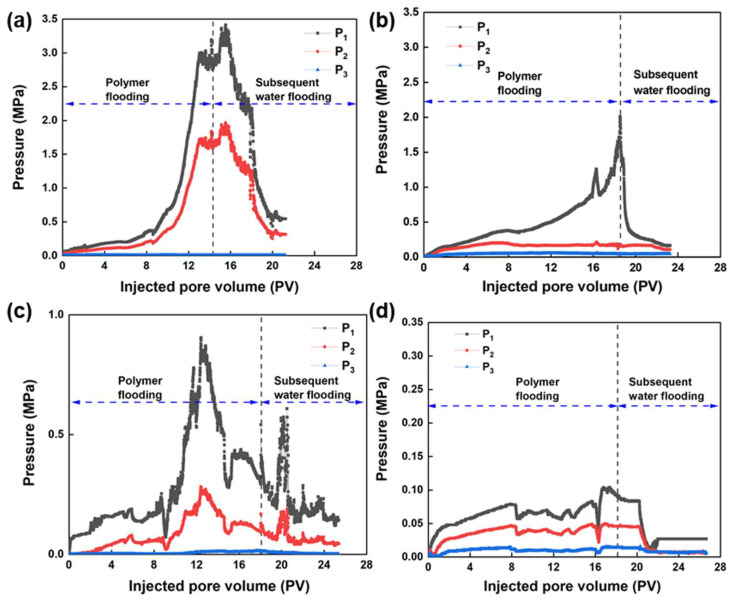
Relationship between the injection pressure and injected pore volume under different ageing times: (**a**) t = 2 h; (**b**) t = 4 h; (**c**) t = 12 h; (**d**) t = 48 h.

The propagation behavior of the controllable phase-transition polymer at different stages in porous media was investigated by conducting sand-pack flow experiments using the core model with multi-point pressure measurements. The relationship between the pressure changes and the injected pore volume at different positions of the core was analyzed to clarify phase-transition behavior, propagation, and plugging capability of the phase-transition polymer at different ageing stages in porous media. The relationship between the pressure and injected pore volume during the percolation process of the phase-transition polymer in porous media depending on ageing time is shown in Figure 5.

**Figure 6:**
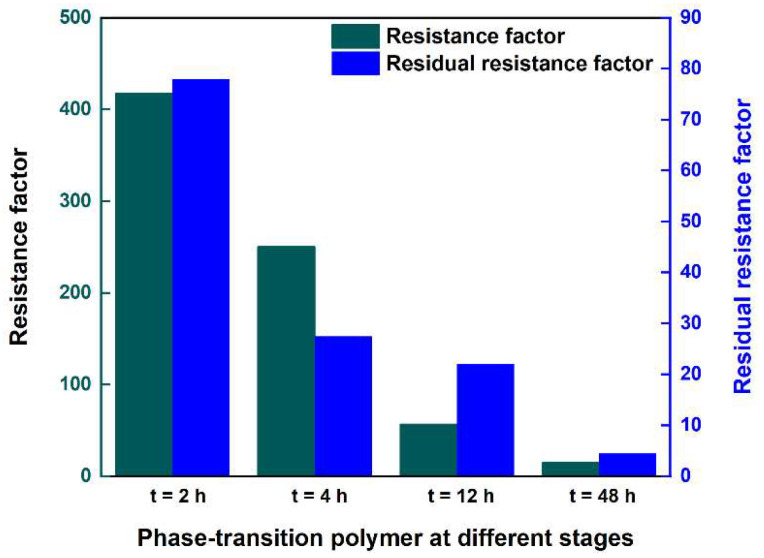
The resistance factor and residual resistance factor of the phase-transition polymer at different ageing times: t = 2 h; t = 4 h; t = 12 h; t = 48 h.

As the ageing of the phase-transition polymer proceeds, the injection pressure during the injection process exhibits a decreasing trend. Moreover, in the subsequent waterflooding process, the pressure decreases to a stable value, and the stable pressure value decreases with the extension of ageing time. The results indicate that the phase-transition polymer changes from a dispersed-phase particle gel to a continuous-phase aqueous polymer solution. In order to quantitatively characterize the propagation characteristics of phase-transition polymers in porous media, the pressure change trend at each pressure measurement point during the injection process of phase-transition polymers at different ageing times was analyzed. According to the pressure changes at different positions of the core, the phase-transition polymer shows favorable propagation capability in porous media. The resistance factor and residual resistance factor of phase-transition polymers at different stages were calculated, as shown in Table 1 and Figure 6. As the ageing time prolongs, the two factors decrease, indicating that the phase-transition polymer changes from a dispersed-phase particle gel to a continuous-phase aqueous polymer solution, which aligns with the previous reports [46].

### 3.4. The EOR Efficiency of the Phase-Transition Polymer

**Figure 7:**
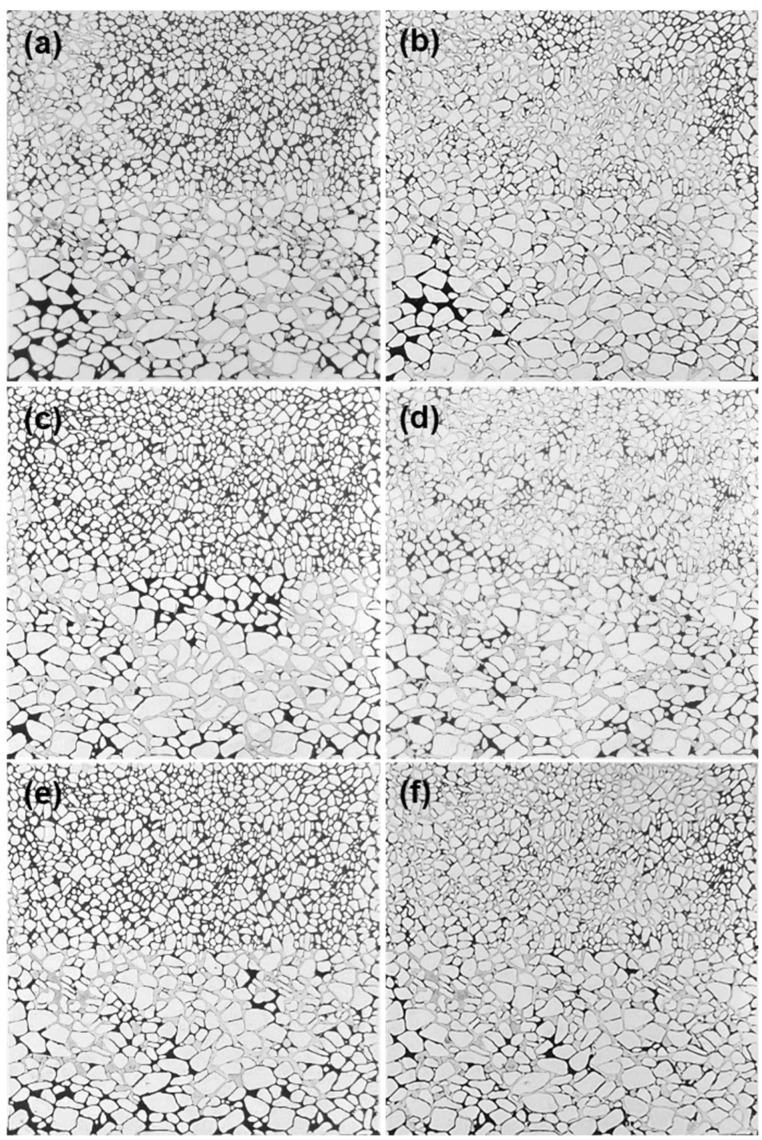
The residual oil distributions after waterflooding and phase-transition polymer flooding at different stages: (**a**) waterflooding and (**b**) polymer flooding—dispersed particle gel stage; (**c**) waterflooding and (**d**) polymer flooding—particle gel–aqueous solution stage; (**e**) waterflooding and (**f**) polymer flooding—continuous-phase aqueous solution stage.

To reveal the EOR mechanism of phase-transition polymer at different stages, three microscopic visualization flooding experiments were carried out. The residual oil distributions after waterflooding and phase-transition polymer flooding at different stages are shown in Figure 7. Results show that due to the heterogeneity of the micro etching model, injected water mainly flows along the high pore throat flow channel during the waterflooding process, which can effectively recover crude oil in the high permeability area. The micro residual oil mainly exists in low permeability areas at the end of waterflooding, and the waterflooding recovery rate is low. After injecting the controllable phase-transition polymers at different stages, the polymers can change the fluid flow direction and expand the sweep efficiency, which can effectively recover the residual oil in low permeability areas. Difference exists in recovering the remaining oil in the low permeability zone of the phase-transition polymer at different stages.

According to the residual oil distribution of phase-transition polymers at different stages, oil recovery at different flooding stages was calculated using the image processing software ImageJ v.153k. The results are summarized in Table 2.

At the initial stage of dispersed-phase particle gel, the controllable phase-transition polymer had low viscosity and a high elastic modulus, which mainly plays a role in blocking and adjusting the liquid flow direction. The increased oil recovery rate was 23.2%. At the stage of the coexistence of the particle gel and aqueous polymer solution, the viscosity of the phase-transition polymer increased rapidly and the particle had deformation and plugging capability, which can play a synergistic role in changing the fluid flow direction via particle plugging and mobility control. The incremental oil recovery was 34.4%. When the controllable phase-transition polymer was in the continuous-phase polymer solution stage, its viscosity reached its maximum, which can play a role in the effect of increasing viscosity and mobility control, followed by an incremental oil recovery of 27.7%.

### 3.5. Enhanced Oil Recovery Capability of Phase-Transition Polymer over Conventional Polymer

#### 3.5.1. Analysis of the Flooding Curves

**Figure 8:**
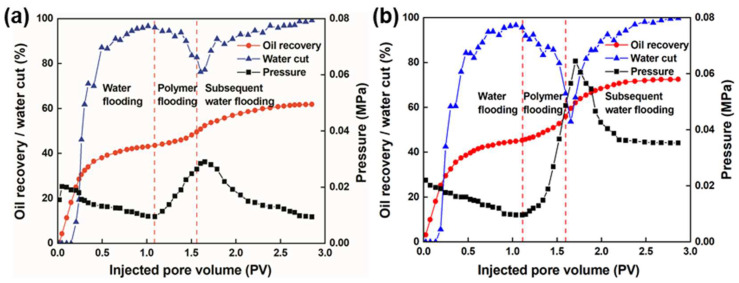
Comparison of the oil flooding curves between (**a**) conventional polymer and (**b**) controllable phase-transition polymer.

Figure 8 presents the flooding curves of the phase-transition polymer over conventional polymer. Results show that during the initial waterflooding stage, when the injection water volume reached around 0.2 PV, water was produced. With an increase of injection pore volume, the water cut gradually increased and the flow resistance decreased. The pressure slowly decreased to gradually stabilize. When the water cut reached around 95%, it fluctuated slightly, and the waterflooding recovery gradually stabilized. At the end of the waterflooding period, the waterflooding recoveries of conventional polymer and phase-transition polymer were 43.5% and 45.3%, respectively.

Then, a 0.5 PV polymer slug was injected into the sand-pack and flow resistance increased, resulting in an increase in the injection pressure and a decrease in the water cut. The polymer slug can block the high permeability sand-pack, diverting the liquid flow to the low permeability sand-pack. In the subsequent waterflooding stage, a small amount of polymer still remained in the pores and continued to function, and the water cut continued to decrease to the lowest level. The subsequent reversal of the liquid flow profile caused the water cut to rise again to around 98%, and the pressure continued to rise to the highest value, followed by decreasing and stabilizing.

#### 3.5.2. Analysis of Fractional Flow Curves

**Figure 9:**
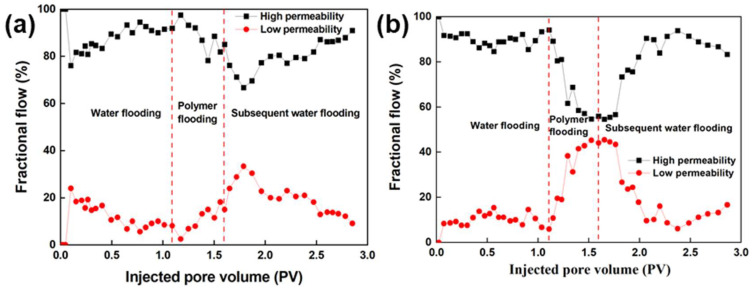
Comparison of fractional flow curves between (**a**) conventional polymer and (**b**) controllable phase-transition polymer.

According to the fractional flow curve in Figure 9, the change in the fractional flow curve of the high- and low-permeability layers at the early stage of waterflooding is not significant. However, after injecting the polymer, the fraction flow of the high-permeability sand-pack rapidly decreases, while the fractional flow of the low permeability sand-pack rapidly increases.

As the injection volume increases, the factional flow curve in the high- and low-permeability layers is the closest, with the fractional flow in the high-permeability layer reaching the lowest and the fractional flow in the low-permeability layer reaching the highest and maintaining fluctuations. During polymer injection, the polymer preferentially flows towards the high-permeability layer, causing an increase in the seepage resistance of the high-permeability layer and then turning towards the low-permeability layer, resulting in a rapid increase in the seepage resistance of the low-permeability layer and then flowing towards the high-permeability layer again, causing a profile reversal. After the subsequent waterflooding, the liquid intake amount in the high-permeability sand-pack starts to increase, while the liquid intake amount in the low-permeability sand-pack starts to decrease, fluctuating up and down to a certain value. During conventional polymer flooding, its fractional flow curve shows a “Λ” shaped change, while a “Ո” shaped change in fractional flow curve was observed during the controllable phase-transition polymer flooding, which indicates that the longer the controllable phase-transition polymer acts in the low-permeability layer, the more obvious the sweep efficiency improves.

#### 3.5.3. Analysis of Enhanced Oil Recovery

**Figure 10:**
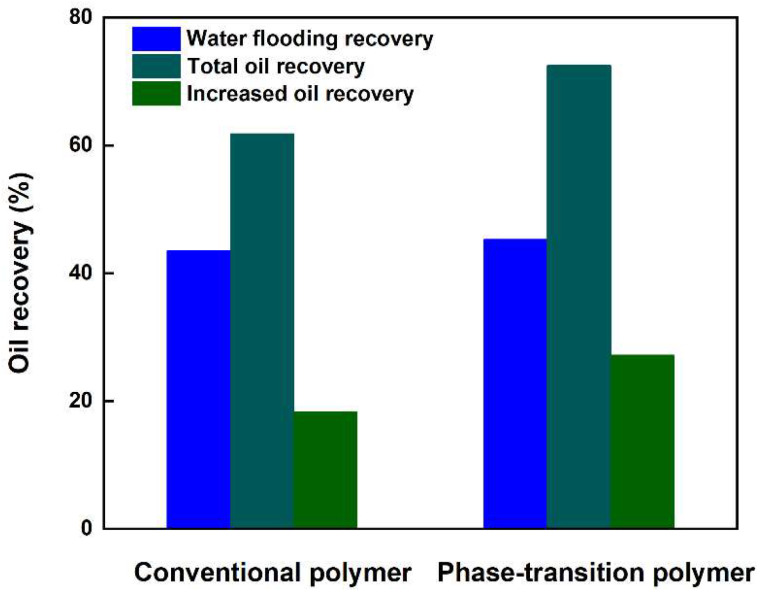
Comparison of enhanced recovery efficiency between conventional polymer and controllable phase transition-polymer.

Figure 10 depicts the results of enhanced recovery efficiency between conventional polymer and controllable phase-transition polymer. The total oil recovery rate is 61.8% and 72.5% for the conventional polymer and controllable phase-transition polymer, respectively. Moreover, the recovery of the novel controllable phase-transition polymer was 27.2% after waterflooding, which was 8.9% higher than that of conventional polymer. This can be attributed to the superior physiochemical properties of the phase transition-polymer, as analyzed above.

## 4. Conclusions

In this study, to reveal the phase-transition behavior, propagation, and oil recovery enhancement mechanism of the novel controllable phase-transition polymer, a series of experiments were systematically conducted to gain mechanistic insights into enhanced oil recovery for mature waterflooding reservoirs. Results show that the controllable phase-transition polymer is in a state of dispersed-phase particle gel at the initial stage before phase transition, and a small amount of dense network structure exists. As the ageing time prolongs, the unique weak cross-linking structure of this polymer is hydrolyzed and transformed into a continuous-phase state of high viscosity polymer solution. Its morphology presents a large number of network structures, which can improve viscosity. With ageing continuing, the viscosity gradually increases and the elastic modulus decreases significantly due to its gradual dissolution in water, forming an intermediate phase when particle gel and polymer solution coexist. At the stabilization stage, the viscosity and elastic modulus of the phase-transition polymer tend to be stable, and are higher than those of conventional HPAM. The controllable phase-transition polymer exhibits favorable phase-transition behavior and good propagation capability in porous media. The oil recovery rate of the controllable phase-transition polymer was highest when the particle gel and polymer solution coexisted, followed by that of a pure continuous-phase polymer solution and pure dispersed-phase particle gel suspension. The recovery rate of the novel controllable phase-transition polymer was 27.2% after waterflooding, which was 8.9% higher than that of conventional polymer.

## Figures and Tables

**Table 1:** The resistance factor and residual resistance factor of the phase-transition polymer.

Ageing Time/h	∆*P*_wa_/MPa	∆*P*_polymer_/MPa	∆*P*_wb_/MPa	*F*_r_	*F*_rr_
2	0.007	2.926	0.546	418.0	78.0
4	0.006	1.503	0.165	250.5	27.5
12	0.0065	0.369	0.143	56.8	22.0
48	0.006	0.092	0.027	15.3	4.5

**Table 2:** Comparison of incremental oil recovery for phase-transition polymer at different flooding stages.

Different Phase Stage	Waterflooding Recovery/%	Polymer Flooding Recovery/%	Increased Oil Recovery/%
Dispersed-phase particle gel	23.6	46.8	23.2
Particle gel–aqueous solution mesophase	24.5	58.9	34.4
Continuous-phase aqueous solution	24.6	52.3	27.7

## Data Availability

Data are contained within the article and supplementary materials.

## Supplementary Materials

The following supporting information can be downloaded at: https://www.mdpi.com/article/10.3390/nano13243101/s1. Figure S1: The schematic diagram of sand-pack flow experimental setup. Figure S2: The schematic diagram of micromodel flooding experimental setup. Figure S3: Digital images of controllable phase-transition polymer at different stages: (a) dispersed-phase particle gel stage, (b) particle gel-aqueous solution mesophase stage and (c) continuous-phase aqueous solution stage.

## References

[1] Kamal M.S., Hussein I.A., Sultan A.S. (2017). Review on surfactant flooding: Phase behavior, retention, IFT, and field applications. Energy Fuels.

[2] Yang W., Lu J., Wei B., Yu H., Liang T. (2021). Micromodel studies of surfactant flooding for enhanced oil recovery: A review. ACS Omega.

[3] Guangzhi L., Qiang W., Hongzhuang W., Weidong L., Zhengmao W. (2017). Chemical flooding development status and prospect. Acta Petrol. Sin..

[4] Wang D., Seright R.S., Shao Z., Wang J. (2008). Key aspects of project design for polymer flooding at the Daqing oilfield. SPE Reserv. Eval. Eng..

[5] Sheng J.J., Leonhardt B., Azri N. (2015). Status of polymer-flooding technology. J. Can. Pet. Technol..

[6] AlSofi A.M., Blunt M.J. (2014). Polymer flooding design and optimization under economic uncertainty. J. Pet. Sci. Eng..

[7] Kamal M.S., Sultan A.S., Al-Mubaiyedh U.A., Hussein I.A. (2015). Review on polymer flooding: Rheology, adsorption, stability, and field applications of various polymer systems. Polym. Rev..

[8] Hirasaki G.J., Miller C.A., Puerto M. (2011). Recent advances in surfactant EOR. SPE J..

[9] Kianinejad A., Ghazanfari M.H., Kharrat R., Rashtchian D. (2013). An experimental investigation of surfactant flooding as a good candidate for enhancing oil recovery from fractured reservoirs using one-quarter five spot micromodels: The role of fracture geometrical properties. Energy Sources Part A.

[10] Ko K.M., Chon B.H., Jang S.B., Jang H.Y. (2014). Surfactant flooding characteristics of dodecyl alkyl sulfate for enhanced oil recovery. J. Ind. Eng. Chem..

[11] Morshedi S., Foroughi S., Beiranvand M.S. (2014). Numerical simulation of surfactant flooding in darcy scale flow. Pet. Sci. Technol..

[12] Alwated B., El-Amin M.F. (2021). Enhanced oil recovery by nanoparticles flooding: From numerical modeling improvement to machine learning prediction. Adv. Geo-Energy Res..

[13] Ahmadi M.A., Shadizadeh S.R. (2017). Nano-surfactant flooding in carbonate reservoirs: A mechanistic study. Eur. Phys. J. Plus.

[14] Zargartalebi M., Kharrat R., Barati N. (2015). Enhancement of surfactant flooding performance by the use of silica nanoparticles. Fuel.

[15] Azarshin S., Moghadasi J., A Aboosadi Z. (2017). Surface functionalization of silica nanoparticles to improve the performance of water flooding in oil wet reservoirs. Energy Explor. Exploit..

[16] Youssif M.I., El-Maghraby R.M., Saleh S.M., Elgibaly A. (2018). Silica nanofluid flooding for enhanced oil recovery in sandstone rocks. Egypt. J. Pet..

[17] Tavakkoli O., Kamyab H., Shariati M., Mustafa Mohamed A., Junin R. (2022). Effect of nanoparticles on the performance of polymer/surfactant flooding for enhanced oil recovery: A review. Fuel.

[18] Druetta P., Picchioni F. (2019). Polymer and nanoparticles flooding as a new method for Enhanced Oil Recovery. J. Pet. Sci. Eng..

[19] Cheraghian G., Hendraningrat L. (2016). A review on applications of nanotechnology in the enhanced oil recovery part B: Effects of nanoparticles on flooding. Int. Nano Lett..

[20] Rueda E., Akarri S., Torsæter O., Moreno R.B. (2020). Experimental investigation of the effect of adding nanoparticles to polymer flooding in water-wet micromodels. Nanomaterials.

[21] Al-Asadi A., Rodil E., Soto A. (2022). Nanoparticles in Chemical EOR: A Review on Flooding Tests. Nanomaterials.

[22] Torsæter O. (2021). Application of nanoparticles for oil recovery. Nanomaterials.

[23] Rai K., Johns R.T., Delshad M., Lake L.W., Goudarzi A. (2013). Oil-recovery predictions for surfactant polymer flooding. J. Pet. Sci. Eng..

[24] Shaker Shiran B., Skauge A. (2013). Enhanced oil recovery (EOR) by combined low salinity water/polymer flooding. Energy Fuels.

[25] Sun C., Guo H., Li Y., Song K. (2020). Recent advances of surfactant-polymer (SP) flooding enhanced oil recovery field tests in China. Geofluids.

[26] Gao C., Shi J., Zhao F. (2014). Successful polymer flooding and surfactant-polymer flooding projects at Shengli oilfield from 1992 to 2012. J. Pet. Explor. Prod. Technol..

[27] Wang H., Cao X., Zhang J., Zhang A. (2009). Development and application of dilute surfactant-polymer flooding system for Shengli oilfield. J. Pet. Sci. Eng..

[28] Ma Y., Hou J., Zhao F., Song Z. (2018). Linearly descending viscosity for alkaline-surfactant-polymer flooding mobility modification in multilayer heterogeneous reservoirs. RSC Adv..

[29] Pal N., Saxena N., Mandal A. (2018). Characterization of alkali-surfactant-polymer slugs using synthesized gemini surfactant for potential application in enhanced oil recovery. J. Pet. Sci. Eng..

[30] Alkhatib A., Babaei M. (2016). Applying the multilevel monte carlo method for heterogeneity-induced uncertainty quantification of surfactant/polymer flooding. SPE J..

[31] Wang Y., Liu H., Wang J., Dong X., Chen F. (2019). Formulation development and visualized investigation of temperature-resistant and salt-tolerant surfactant-polymer flooding to enhance oil recovery. J. Pet. Sci. Eng..

[32] Al-Murayri M.T., Hassan A.A., Abdullah M.B., Abdulrahim A.M., Marlière C., Hocine S., Tabary R., Suzanne G.P. (2019). Surfactant/polymer flooding: Chemical-formulation design and evaluation for Raudhatain lower Burgan Reservoir, Kuwait. SPE Reservoir Eval. Eng..

[33] Aramideh S., Borgohain R., Naik P.K., Johnston C.T., Vlachos P.P., Ardekani A.M. (2018). Multi-objective history matching of surfactant-polymer flooding. Fuel.

[34] Abdulbaki M., Huh C., Sepehrnoori K., Delshad M., Varavei A. (2014). A critical review on use of polymer microgels for conformance control purposes. J. Pet. Sci. Eng..

[35] He H., Fu J., Hou B., Yuan F., Guo L., Li Z., You Q. (2018). Investigation of injection strategy of branched-preformed particle gel/polymer/surfactant for enhanced oil recovery after polymer flooding in heterogeneous reservoirs. Energies.

[36] Liu W., He H., Yuan F., Liu H., Zhao F., Liu H., Luo G. (2022). Influence of the injection scheme on the enhanced oil recovery ability of heterogeneous phase combination flooding in mature waterflooded reservoirs. ACS Omega.

[37] He H., Liu W., Chen Y., Liu H., Liu H., Luo G. (2022). Synergistic mechanism of well pattern adjustment and heterogeneous phase combined flooding on enhancing oil recovery in mature fault-block reservoirs. J. Pet. Explor. Prod. Technol..

[38] Wu D., Zhou K., Hou J., An Z., Zhai M., Liu W. (2020). Experimental study on combining heterogeneous phase composite flooding and streamline adjustment to improve oil recovery in heterogeneous reservoirs. J. Pet. Sci. Eng..

[39] Zhang X., Zhang Y., Liu H., Li S., Liu L. (2023). Dynamic sweep experiments on a heterogeneous phase composite system based on branched-preformed particle gel in high water-cut reservoirs after polymer flooding. Gels.

[40] Jung J.C., Zhang K., Chon B.H., Choi H.J. (2013). Rheology and polymer flooding characteristics of partially hydrolyzed polyacrylamide for enhanced heavy oil recovery. J. Appl. Polym. Sci..

[41] He H., Chen Y., Yu Q., Wen X., Liu H. (2019). Optimization design of injection strategy for surfactant-polymer flooding process in heterogeneous reservoir under low oil prices. Energies.

[42] Ji Y., Yang X., Ji Z., Zhu L., Ma N., Chen D., Jia X., Tang J., Cao Y. (2020). DFT-calculated IR spectrum amide I, II, and III band contributions of n-methylacetamide fine components. ACS Omega.

[43] Yang J.C., Jablonsky M.J., Mays J.W. (2002). NMR and FT-IR studies of sulfonated styrene-based homopolymers and copolymers. Polymer.

[44] Yao S., Beginn U., Gress T., Lysetska M., Würthner F. (2004). Supramolecular polymerization and gel formation of bis(merocyanine) dyes driven by dipolar aggregation. J. Am. Chem. Soc..

[45] Katashima T. (2021). Rheological studies on polymer networks with static and dynamic crosslinks. Polym. J..

[46] Zhu S., Zhang S., Xue X., Zhang J., Xu J., Liu Z. (2022). Influencing factors for effective establishment of residual resistance factor of polymer solution in porous media. J. Polym. Res..

