# Supplementary material for: Mechanistic Insights into a Novel Controllable Phase-Transition Polymer for Enhanced Oil Recovery in Mature Waterflooding Reservoirs
Source: Nanomaterials (Basel). 2023 Dec 8;13(24):3101. doi: 10.3390/nano13243101 (PMC10745705; doi:10.3390/nano13243101)
Supplement: Supplementary file 1 [file nanomaterials-13-03101-s001.zip › nanomaterials-2706147-supplementary.pdf]

Mechanistic Insights into a Novel Controllable Phase-Transition Polymer for Enhanced Oil Recovery in Mature Waterflooding Reservoirs

Yong Yang [1,2], Xiaopeng Cao [1], Yanfeng Ji [1] and Ruqiang Zou [2,*]

[1] Shengli Oilfield, SINOPEC, Dongying 257001, China

[2] School of Materials Science and Engineering, Peking University, Beijing 100871, China

*Correspondence: rzou@pku.edu.cn

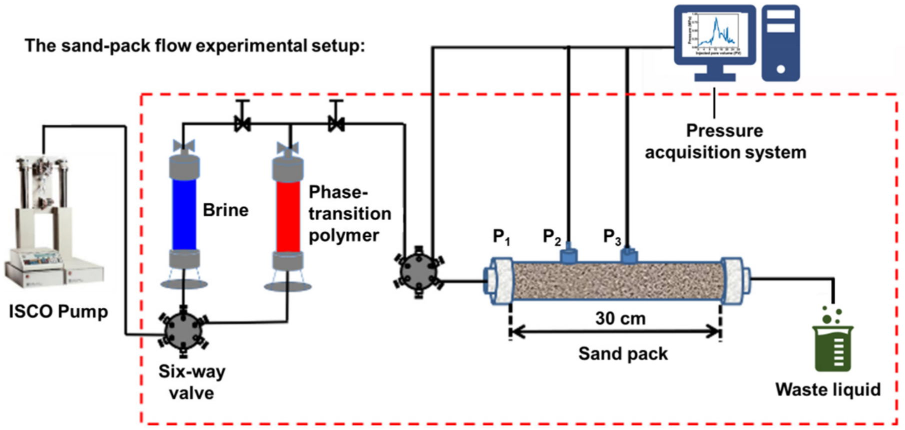

Figure S1. The schematic diagram of sand-pack flow experimental setup.

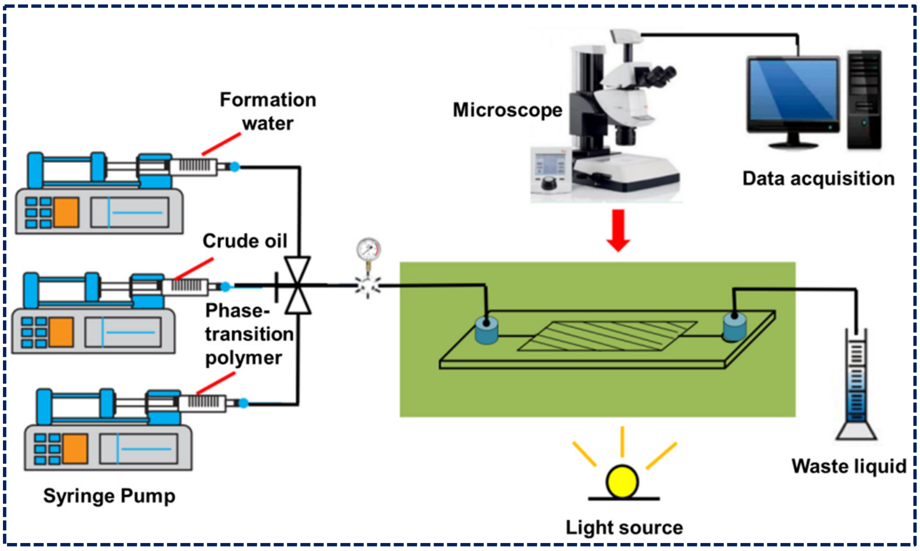

Figure S2. The schematic diagram of micromodel flooding experimental setup.

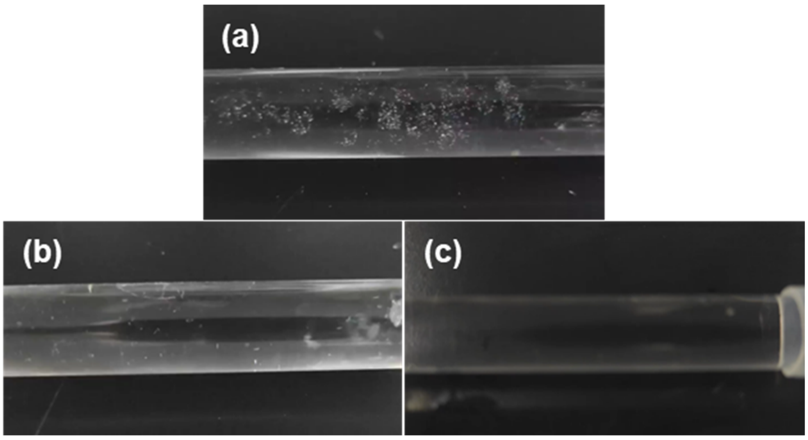

Figure S3. Digital images of controllable phase-transition polymer at different stages: (a) dispersed-phase particle gel stage, (b) particle gel-aqueous solution mesophase stage and (c) continuous-phase aqueous solution stage.
